# Correlation between body mass index (BMI) and the Gleason score of prostate biopsies in Chinese population

**DOI:** 10.18632/oncotarget.11453

**Published:** 2016-08-20

**Authors:** Feng Zhou, Xi Chen, Jinxian Pu, Jun Ouyang, Gang Li, Jigen Ping, Yong Lu, Jianquan Hou, Yong Han

**Affiliations:** ^1^ Department of Urology, The First Affiliated Hospital of Soochow University, Suzhou, Jiangsu, P. R. China; ^2^ VIP center, Zhejiang Provincial People's Hospital, Hangzhou, Zhejiang, P. R. China; ^3^ Department of Pathology, Zhejiang Provincial People's Hospital, Hangzhou, Zhejiang, P. R. China

**Keywords:** prostate cancer, Gleason score, BMI

## Abstract

We assessed the correlation between BMI and Gleason score in prostate biopsies in Chinese Population. In this retrospective study, we collected the Gleason score, PSA, BMI, age, race, and other related clinical data on 290 patients who had undergone prostatic biopsy. We then compared the prostate cancer detection rates and Gleason scores between the high BMI group (BMI ≥ 25; 143 cases) and low BMI group (< 25; 147 cases). Among the 137 patients in whom prostate cancer detected, 70 had high BMIs and 67 had normal BMIs, making the detection rates 48.95% and 45.58% respectively. Seventeen prostate cancer patients had low Gleason scores (Gleason score < 7), while 120 had high Gleason scores (≥ 7). Within the high BMI group, 44.76% had high Gleason scores, which was significantly greater than the 38.10% in the low BMI group (*P* = 0.027). These results indicate that while there was no effect of BMI on the rate of positive prostate cancer biopsies, the rate of high Gleason scores was greater in the high BMI group than the normal BMI group.

## INTRODUCTION

Prostate cancer (PCa) is the most common malignant tumor of the male urinary system. In recent years, the mortality rates for PCa have increased globally, except in a few high-income countries such as United States [1–3]. With the popularization and application of prostate-specific antigen (PSA) screening, a growing number of patients are receiving prostate needle biopsies to confirm PCa. Body mass index (BMI) is an independent risk factor for hypertension, diabetes and coronary heart disease, among others [4–7]. To investigate the relationship between PCa and BMI, between February 2012 and January 2014, we measured the BMIs of 290 patients at our hospital and assessed the clinical significance of BMI with respect to prostate biopsy in Chinese Population.

## RESULTS

### General characteristics of the study population

Among the 290 patients studied, the biopsies of 137 were diagnosed as PCa and 153 were determined to be benign prostate tissue. Total PSA (tPSA), free-to-total PSA ratios (f/t ratios) and total prostate volume (TPV) all significantly differed (*P* < 0.001) between the PCa and benign groups. Likewise, there were significant differences between the PCa and benign groups with respect to median tPSA (PCa: 42.7 ng/ml vs. benign: 13.5 ng/ml) and corresponding f/t ratios (PCa: 0.11 vs. benign: 0.16) and TPV (PCa: 35 cm^3^ vs. benign 51 cm^3^) (Table 1).

**Table 1:** Clinical data for patients with benign prostate tissue and PCa

	PCa (137)	Benign (153)	*P*
Age (years)	74.12 ± 7.60	71.40 ± 8.14	0.03
tPSA (ng/ml)	42.7 (2.99 – > 100)	13.5 (8.4–21)	< 0.001
f/t ratio	0.11 (0.08–0.16)	0.16 (0.1–0.21)	< 0.001
TPV (cm3)	35 (21–73)	51 (33–81)	< 0.001

Values are expressed as mean ± standard deviation. Total prostate-specific antigen (tPSA), free-to-total prostate specific antigen ratio (f/t ratio) and total prostate volume (TPV).

### Analysis and comparison of BMI in PCa and benign groups

Among the 143 patients with a high BMI (≥ 25), PCa was detected in 70. Among the 147 patients with a low BMI (< 25), PCa was detected in 67. The detection rates were thus 48.95% and 45.58% for the high and low BMI groups, respectively, which was not a significant difference (Table 2).

**Table 2:** Analysis and comparison of BMI in the PCa and Benign groups

	PCa (137)	Benign (153)	All	Rate (%)
High BMI group	70	73	143	49.0
Low BMI group	67	80	147	45.6
All	137	153	290	

*P* > 0.05 (PCa rate in high BMI group vs. low BMI group).

### A comparative analysis of the relationship between Gleason score and BMI in patients with PCa

Seventeen of the PCa patients had a low Gleason score (< 7), while 120 had a high Gleason score (≥ 7 points). Patients with a high Gleason score accounted for 44.76% (64 cases) of those in the high BMI group, which was significantly higher than the 38.10% (56 cases) in the low BMI group (*P* < 0.05) (Table 3).

**Table 3:** Analysis of the relationship between Gleason score and BMI in patients with PCa

	All	PCa	High Gleason group	HGPCa rate
High BMI group	143	70	64	44.8%
Low BMI group	147	67	56	38.1%
All	290	137	120	

*P* = 0.027 (HGPCa rate in high BMI group vs. low BMI group).

## DISCUSSION

Accompanying the development of modern society and the improvement of living standards is the increasing incidence of obesity. According to statistics gathered in developed countries, the obesity rate among adults has reached 35%, and 15% to 20% patients die from various diseases combined with obesity [8]. Consequently, the relationship between obesity and disease is receiving more and more attention. Obesity is often accompanied by three conditions, high blood pressure, high blood sugar, and high serum cholesterol or triglyceride levels, which in combination are referred to as metabolic syndrome [9, 10]. PCa is the most important threat to male health in many European countries, and it has the highest incidence of cancer among males in the United States, and is the second highest cause of death among males [11–14]. With improvements in people's living standards and advances in medical diagnosis, the incidence of PCa in China is increasing each year [15–19]. Epidemiological studies have shown that obesity increases the prevalence and mortality of multiple cancers, and increasing evidence suggests obesity is associated with a high Gleason score for PCa (HGPCa) and an increase in PCa-specific mortality [20, 21]. We found that although BMI is not an independent risk factor for the development of PCa, it is associated with HGPCa. These results are consistent with an earlier study of 4,926 American patients by Kryvenko et al. [22]. The samples collected by Kryvenko et al. were from Western world patients and they may differ from Asian population in several clinicopathological aspects. Moreover, since there are no such extensive PSA screening in China as in the Western world, PSA values measured in this study are strikingly different from what we currently see in the Western world patients. Based on these points, our research will be a valuable addition to their work.

In this retrospective study of patients who underwent prostate biopsy, 44.8% of those with a high BMI also had a high Gleason score, which was significantly higher than the fraction in the low BMI group (38.1%) (*P* = 0.027). This suggests obese patients will increase the HGPCa-positivity rate for prostate punctures. Liang et al. performed a logistic regression, adjusting for age, race, rectal examination, family history of PCa, past medical history, etc. [7]. They found that BMI is positively associated with PCa and high HGPCa. In addition, Lee et al. observed that BMIs in Asian men correlated with the results of prostate biopsy [23]. Our results also showed that the rate of positive prostate biopsies is associated with age, tPSA, f/t ratio and TPV, but more importantly, we found that BMI significantly affected the HGPCa rate among prostate biopsies. We therefore suggest that in clinical settings, in order to detect possible HGPCa through prostate puncture, the BMI should be considered as well as the age, tPSA, f/t ratio and TPV.

A BMI provides predictive information about the risk of HGPCa and is an important factor affecting the Gleason score of prostate biopsies. Nevertheless, because of the small sample size in this study, its statistical power and our ability to draw conclusions are limited. More patients from our center as well as other hospitals should be recruited in future studies.

## MATERIALS AND METHODS

### Materials

We studied a group of 290 patients with an average age of 72.5 years (range, 39 to 89 years). Serum tPSA was > 10 ng/mL in 203 cases, 4 to 10 ng/mL in 72 cases, and < 4 ng/mL in 15 cases. All of the patients underwent rectal ultrasound prostate biopsy, after which 137 samples were diagnosed as pathological PCa, and 153 samples were determined to be benign prostate tissue. Biopsy specimens containing adenocarcinoma were scored according to the Gleason grading system, and HGPCa was defined as the presence of a Gleason score ≥ 4 + 3.

### Research methods

#### Determination of serum tPSA, TPV, f/t ratios and BMI

Serum tPSA was determined using a chemiluminescence method. Total prostate volume (TPV) was calculated as: 0.52 × Left and right diameter × anteroposterior diameter × upper and lower diameters. f/t ratios were calculated as: serum free PSA/serum tPSA. BMI was calculated as: body weight (kg) / height^2^ (m)

#### Ultrasound guided prostate biopsy

Using a Sequoia 512 color Doppler ultrasound diagnostic system (Siemens Company). Prostate needle biopsies were carried out using an 18 G needle and an automated biopsy gun (Bard Inc.). Prostate needle biopsies were performed at 12 sites, and suspicious lesions were sampled 1–3 times. Six sites each were punctured in the left and right lobes of the prostate. Suspicious lesions were punctured within prostate nodules and abnormal echo areas, after which the biopsy specimens were sent for pathological examination. To prevent infection, antibiotics were used for 3 days.

### Statistical methods

SPSS 17 software was used for statistical analysis. TPSA, f/t ratio, TPV and other information with skewed distributions were expressed as median and quartiles (P25 to P75). Normally distributed data were described as the mean ± standard deviation. In the two groups, normally distributed data were compared using *t* tests. Rates were compared using the χ^2^ test. Values of *P* < 0.05 were considered significant.

## References

[1] Torre LA, Bray F, Siegel RL, Ferlay J, Lortet-Tieulent J, Jemal A (2015). Global cancer statistics, 2012. Ca-Cancer J Clin.

[2] Center MM, Jemal A, Lortet-Tieulent J, Ward E, Ferlay J, Brawley O, Bray F (2012). International variation in prostate cancer incidence and mortality rates. Eur Urol.

[3] Zhou CK, Check DP, Lortet-Tieulent J, Laversanne M, Jemal A, Ferlay J, Bray F, Cook MB, Devesa SS (2016). Prostate cancer incidence in 43 populations worldwide: An analysis of time trends overall and by age group. Int J Cancer.

[4] Discacciati A, Orsini N, Wolk A (2012). Body mass index and incidence of localized and advanced prostate cancer—a dose-response meta-analysis of prospective studies. Ann Oncol.

[5] Bonn SE, Sjolander A, Tillander A, Wiklund F, Gronberg H, Balter K (2016). Body mass index in relation to serum prostate-specific antigen levels and prostate cancer risk. Int J Cancer.

[6] Renehan AG, Tyson M, Egger M, Heller RF, Zwahlen M (2008). Body-mass index and incidence of cancer: a systematic review and meta-analysis of prospective observational studies. Lancet.

[7] Liang YY, Ketchum NS, Goodman PJ, Klein EA, Thompson IM (2014). Is There a Role for Body Mass Index in the Assessment of Prostate Cancer Risk on Biopsy?. J Urology.

[8] Isbarn H, Jeldres C, Budaus L, Salomon G, Schlomm T, Steuber T, Chun FK, Ahyai S, Capitanio U, Haese A, Heinzer H, Huland H, Graefen M, Karakiewicz P (2009). Effect of body mass index on histopathologic parameters: results of large European contemporary consecutive open radical prostatectomy series. Urology.

[9] Tyson MD, Arora SS, Scarpato KR, Barocas D (2016). Magnetic resonance-ultrasound fusion prostate biopsy in the diagnosis of prostate cancer. Urol Oncol.

[10] Wang SY, Shiboski S, Belair CD, Cooperberg MR, Simko JP, Stoppler H, Cowan J, Carroll PR, Blelloch R (2014). miR-19, miR-345, miR-519c-5p serum levels predict adverse pathology in prostate cancer patients eligible for active surveillance. Plos One.

[11] Hu MB, Liu SH, Jiang HW, Bai PD, Ding Q (2014). Obesity affects the biopsy-mediated detection of prostate cancer, particularly high-grade prostate cancer: a dose-response meta-analysis of 29,464 patients. Plos One.

[12] Gong Z, Neuhouser ML, Goodman PJ, Albanes D, Chi C, Hsing AW, Lippman SM, Platz EA, Pollak MN, Thompson IM, Kristal AR (2006). Obesity, diabetes, and risk of prostate cancer: results from the prostate cancer prevention trial. Cancer Epidem Biomar.

[13] Chu DI, De Nunzio C, Gerber L, Thomas JA, Calloway EE, Albisinni S, Senocak C, McKeever MG, Moreira DM, Tubaro A, Moul JW, Freedland SJ, Banez LL (2011). Predictive value of digital rectal examination for prostate cancer detection is modified by obesity. Prostate Cancer P D.

[14] Luo Y, Gou X, Huang P, Mou C (2014). Prostate cancer antigen 3 test for prostate biopsy decision: a systematic review and meta analysis. Chinese Med J-Pekin.

[15] Bhindi B, Kulkarni GS, Finelli A, Alibhai SMH, Hamilton RJ, Toi A, van der Kwast TH, Evans A, Hersey K, Jewett MAS, Zlotta AR, Trachtenberg J, Fleshner NE (2014). Obesity Is Associated with Risk of Progression for Low-risk Prostate Cancers Managed Expectantly. Eur Urol.

[16] Bjurlin MA, Meng X, Le Nobin J, Wysock JS, Lepor H, Rosenkrantz AB, Taneja SS (2014). Optimization of prostate biopsy: the role of magnetic resonance imaging targeted biopsy in detection, localization and risk assessment. J Urology.

[17] Moller H, Roswall N, Van Hemelrijck M, Larsen SB, Cuzick J, Holmberg L, Overvad K, Tjonneland A (2015). Prostate cancer incidence, clinical stage and survival in relation to obesity: a prospective cohort study in Denmark. Int J Cancer.

[18] Yashi M, Mizuno T, Yuki H, Masuda A, Kambara T, Betsunoh H, Abe H, Fukabori Y, Muraishi O, Suzuki K, Nakazato Y, Kamai T (2014). Prostate volume and biopsy tumor length are significant predictors for classical and redefined insignificant cancer on prostatectomy specimens in Japanese men with favorable pathologic features on biopsy. Bmc Urol.

[19] Kryvenko ON, Diaz M, Matoso A, Kates M, Cohen J, Swanson GP, Epstein JI (2016). Prostate-specific Antigen Mass Density-A Measure Predicting Prostate Cancer Volume and Accounting for Overweight and Obesity-related Prostate-specific Antigen Hemodilution. Urology.

[20] Miyoshi Y, Furuya M, Teranishi J, Noguchi K, Uemura H, Yokomizo Y, Sugiura S, Kubota Y (2014). Comparison of 12- and 16-core prostate biopsy in japanese patients with serum prostate-specific antigen level of 4.0–20.0 ng/mL. Urol J.

[21] Katz DJ, Pinochet R, Richards KA, Godoy G, Udo K, Nogueira L, Cronin AM, Fine SW, Scardino PT, Coleman JA (2014). Comparison of transperineal mapping biopsy results with whole-mount radical prostatectomy pathology in patients with localized prostate cancer. Prostate Cacner.

[22] Kryvenko ON, Epstein JI, Meier FA, Gupta NS, Menon M, Diaz M (2015). Correlation of High Body Mass Index With More Advanced Localized Prostate Cancer at Radical Prostatectomy Is Not Reflected in PSA Level and PSA Density but Is Seen in PSA Mass. Am J Clin Pathol.

[23] Lee A, Chia SJ (2015). Prostate cancer detection: The impact of obesity on Asian men. Urol Oncol.

